# Anti‐Cancer Effect of Sulforaphane in Human Pancreatic Cancer Cells Mia PaCa‐2

**DOI:** 10.1002/cnr2.70074

**Published:** 2024-12-04

**Authors:** Min Ju Park, Yoon Hee Kim

**Affiliations:** ^1^ Department of Food and Nutrition, College of Engineering Daegu University Gyeongsan‐Si Gyeongsangbuk‐do Republic of Korea

**Keywords:** anticancer, apoptosis, GSK‐3β, pancreatic cancer, sulforaphane

## Abstract

**Background:**

Pancreatic cancer is difficult to treat early as it has no early symptoms. The presence of sulforaphane (SFN) in cruciferous vegetables has been found to possess anti‐cancer effects in gastric and colon cancers. Glycogen synthase kinase‐3 beta (GSK‐3β), a serine/threonine kinase, plays a significant role in pancreatic cancer progression, influencing tumor growth, metastasis, and treatment resistance. Targeting GSK‐3β has shown potential to enhance the efficacy of chemotherapy. However, the mechanism underlying the anticancer effects of SFN on pancreatic cancer through GSK‐3β is unclear.

**Aims:**

In this study, we examined the anticancer effects of SFN in human pancreatic cancer cell line Mia PaCa‐2 and evaluated its molecular mechanisms with respect to the GSK‐3β‐related pathway.

**Methods and Results:**

SFN increased the protein expression of the phosphorylated form of GSK3β (Ser9). In the Wingless Int‐1 homolog/β‐catenin pathway, GSK3β induced apoptosis by phosphorylating β‐catenin. However, in mutant Kirsten rat sarcoma viral oncogene homolog‐like‐dependent cells such as Mia PaCa‐2, GSK3β was suppressed and the β‐catenin level was increased, thus inducing apoptosis. Indeed, SFN increased the protein expression of β‐catenin in the cytoplasm and nucleus. Subsequently, we measured the level of cMyc, the target gene of β‐catenin. SFN decreased cMyc expression despite an increase in the β‐catenin. We measured the expression of nuclear factor (NF)‐κB, a downstream factor of GSK3β and an upstream factor of cMyc. SFN decreased the expression of NF‐κB and cMyc, indicating that SFN inhibits cell proliferation by suppressing the GSK3β/NF‐κB/cMyc pathway. As the suppression of NF‐κB results in a decrease in B‐cell lymphoma 2 (BCL‐2) which is the anti‐apoptotic gene, we tested the effect of SFN in the expression of BCL‐2. SFN inhibited the expression of BCL‐2 and increased the ratio of the apoptotic regulator gene BCL‐2 associated X (BAX), where SFN induced the cleaved cysteine aspartase‐3 and poly‐adenosine diphosphate ribose polymerase.

**Conclusion:**

These results indicate that SFN may have therapeutic potential in the inhibition of pancreatic cancer.

## 1 Introduction

According to the 2020 current status report on the incidence of main cancer types by the National Cancer Information Center, pancreatic cancer ranks eighth in terms of commonality, with a crude incidence rate of 16.4%. The mortality of pancreatic cancer, however, is ranked fifth, and the 5‐year relative survival is low at 15.2% compared to that of other cancer types in both males and females [1]. The high mortality and low survival may be due to the lack of biological markers or radiological tests for early diagnosis, leading to over 80% of pancreatic cancer being diagnosed as a cancer that prevents radical resection. Chemotherapy is thus crucial in the treatment of pancreatic cancer [2]. Gemcitabine has been applied in standard systemic chemotherapy since the 1990s; however, numerous ongoing studies are investigating ways to overcome the clinical limitations of monotherapy [2, 3]. The high toxicity of chemotherapy also entails various side effects such as loss of appetite, pain, fatigue, to sleep disorders [4]. Hence, new compounds with positive effects in pancreatic cancer and natural materials with few side effects and low cytotoxicity on healthy cells need to be explored.

Sulforaphane (SFN) is an isothiocyanate, found in the form of glucoraphanin in various cruciferous vegetables such as kale, brussels sprouts, broccoli, and cauliflower [5, 6]. It exhibits antioxidant [7], anti‐inflammatory [8], and anticancer [9] effects. Specifically, previous research on the Mia PaCa‐2 pancreatic cancer cell line has shown that SFN suppresses the expressions of heat shock protein 90 (Hsp90) and its associated proteins, including Akt, cyclin‐dependent kinase 4 (CDK4), and p53, while stimulating the activity of cysteine aspartase (caspase)‐3 to exhibit anti‐cancer effects [10]. Roy et al. demonstrated that SFN induces cell cycle arrest and apoptosis through the regulation of FOXO transcription factors. Pharmacological and genetic inhibitions of PI3K/AKT and MEK/ERK pathways can have synergistic effects on the activation of FOXO transcription factors through dephosphorylation and nuclear retention. Therefore, SFN appears to be an attractive agent for pancreatic cancer prevention and treatment [11]. SFN abrogated resistance by interfering with nuclear factor (NF)‐κB binding in highly treatment‐resistant tumor‐initiating cells such as Mia PaCa‐2 cells [12].

Glycogen synthase kinase 3β (GSK‐3β), a serine/threonine kinase, is a critical player in pancreatic cancer progression, influencing tumor growth, metastasis, and resistance to therapy. GSK‐3β promotes pancreatic cancer, including the regulation of cell proliferation, apoptosis, epithelial‐mesenchymal transition (EMT), and stemness [13, 14]. GSK‐3β could enhance the efficacy of chemotherapy and radiation therapy in pancreatic cancer treatment [13]. These underscores GSK‐3β as a promising target for pancreatic cancer therapy. However, the effect of SFN on GSK‐3β‐ related pathway in Mia PaCa‐2 cells remains unclear. Therefore, in this study, we examined the effect of SFN on the growth and apoptosis of Mia PaCa‐2 cells through GSK‐3β.

## 2 Methods

### 2.1 Materials

SFN was purchased from Penielbio (Daegu, Korea). The concentration of SFN was adjusted to 100 μM using dimethyl sulfoxide (DMSO) (Junsei, Japan). Dulbecco's modified Eagle medium (DMEM), antibiotic antimycotic solution, and 0.25% trypsin‐ethylenediaminetetraacetic acid (EDTA) used for cell culture were purchased from HyClone (Logan, UT, USA), and fetal bovine serum (FBS) was purchased from Atlas Biologicals (Fort Collins, CO, USA). Cell titer 96 Aqueous One Solution cell proliferation assay kit used for cell viability was purchased from Promega (Madison, WI, USA), and The Muse Annexin V & Dead Cell kit used for apoptosis was purchased from Luminex (Austin, TX, USA). Nuclear and cytoplasmic extraction post‐exposure prophylaxis (NE‐PEP) reagents kit used to separate cytoplasm and nucleus was purchased from Thermo Fisher Scientific (Bonn, Germany). The Bio‐Rad protein assay kit used for protein quantification was purchased from Bio‐Rad (Hercules, CA, USA). Enhanced chemiluminescence (ECL) solution for measuring Western blot bands was purchased from ATTO Corporation (Tatio, Tokyo, Japan).

### 2.2 Cell Line and Cell Cultures

Mia PaCa‐2 cells, a human pancreatic cancer cell line, were purchased from Korea Cell Line Bank (Seoul, Korea). Cells were grown in DMEM containing 10% FBS and 1% antibiotic antimycotic solution. Cells were subcultured every 2 or 3 days in a CO_2_ incubator (PHCBi, Wood Dale, IL, USA) adjusted to 37°C and 5% CO_2_.

### 2.3 Cell Viability Assay

To measure the effect of SFN on the cell viability of Mia PaCa‐2 cells, the cells were seeded in a 96‐well plate at a cell count of 1 × 10^4^ cells/200 μL/well. Cells were cultured for 24 h at 37°C in an incubator supplied with 5% CO_2_. Then, the medium was replaced with a medium containing SFN at concentrations of 0, 10, 25, 50, and 100 μM. After 24, 48, or 72 h, the cell proliferation was measured using the Cell titer 96 Aqueous One Solution Cell Proliferation assay kit (Promega) at 490 nm with a microplate reader (Tecan Sunrise‐Basic, Tecan GmbH, Austria). 5‐Fluorouracil (5‐FU) was used as a positive control.

### 2.4 Apoptosis Assay

To measure the effect of SFN on the apoptosis of Mia PaCa‐2 cells, the cells were seeded in a 24‐well plate at a cell count of 5 × 10^4^ cells/mL/well. Cells were cultured for 24 h at 37°C in an incubator supplied with 5% CO_2_. Then, the medium was replaced with a medium containing SFN at concentrations of 0, 25, and 100 μM. Apoptosis was measured 24 h later. Cells were stained with The Muse Annexin V & Dead Cell kit (Luminex, Austin, TX, USA) and measured using a Guava Muse Cell Analyzer (Luminex).

### 2.5 Western Blot Analysis

To measure the effect of SFN on the expression of proteins related to cell proliferation and apoptosis of Mia PaCa‐2 cells, the cells were seeded in a 6‐well plate at a cell count of 4 × 10^4^ cells/3 mL/well. Cells were cultured for 24 h at 37°C in an incubator supplied with 5% CO_2_. Subsequently, the medium was replaced with a medium containing SFN at concentrations of 0, 25, and 100 μM. Cell lysates were obtained 24 h later using cell lysis buffer. Protein concentration was quantified using a Bio‐Rad protein assay kit (Bio‐Rad) standardized to bovine serum albumin (BSA). After adjusting the protein to the same amount, 2 × sodium dodecyl sulfate (SDS) sample buffer was added and heated at 95°C for 5 min. After making a 10%, 12%, or 15% SDS polyacrylamide gel, 20 μL of the same amount of protein sample was added to each well and subjected to electrophoresis. Then, it was transferred to 0.45 μm Immobilon‐P transfer membranes (Merk Millipore, Darmstadt, Germany). After transfer, it was blocked in a 2.5% BSA blocking buffer for 1 h. After blocking, primary antibodies such as anti‐glycogen synthase kinase‐3 beta (GSK‐3β; #5558, 1:1000, Cell Signaling Technology, Beverly, MA, USA), phosphorylated (p‐)GSK‐3β (Ser9; #12456, 1:1000, Cell Signaling), phosphorylated (p‐)NF‐κB p65 (#3033, 1:1000, Cell Signaling), NF‐κB p65 (#8242, 1:1000, Cell Signaling), NF‐κB p50 (E‐10; sc‐8414, 1:200, Santa Cruz Biotechnology, CA, USA), B‐cell lymphoma 2 (BCL‐2; sc‐130 307, 1:200, Santa Cruz), glyceraldehyde 3‐phosphate dehydrogenase (GAPDH; sc‐47 724, 1:200, Santa Cruz), BCL‐2 associated X (BAX; K001593, 1:500, Solarbio Life Sciences, Beijing, China), caspase‐3 (sc‐271 028, 1:100, Santa Cruz), poly‐adenosine diphosphate ribose polymerase (PARP; #9542, 1:1000, Cell Signaling), β‐catenin (#9562, 1:1000, Cell Signaling), cMyc (K002749; 1:500, Solarbio Life Sciences), lamin A/C (#4777, 1:2000, Cell Signaling), and β‐actin (K200058M, 1:5000, Solarbio Life Sciences) were treated and stored overnight at 4°C. After washing four times for 10 min each with Tris‐buffered saline containing 0.1% Tween‐20 (TBST; pH 7.6), secondary antibodies, horseradish peroxidase (HRP)‐conjugated anti‐rabbit immunoglobulin gamma (IgG; #7074, 1:2000, Cell Signaling) or HRP‐conjugated anti‐mouse IgG (#7076, 1:2000, Cell Signaling), were treated and cultured for 1 h and 30 min, respectively. After washing again with TBST, protein bands were measured using VLBER Smart Imaging (Marne‐la‐Vallee, France) with ECL solution (ATTO). The intensity of the bands was analyzed using Image J software (National Institutes of Health, Bethesda, MD, USA).

### 2.6 Separation of Cytoplasmic and Nuclear Proteins

To measure the effect of SFN on β‐catenin in the cytoplasm and nucleus of Mia PaCa‐2 cells, the cells were seeded at a cell count of 1 × 10^6^ cells/5 mL/well. Cells were cultured for 24 h at 37°C in an incubator supplied with 5% CO_2_. Then, the medium was replaced with a medium containing SFN at concentrations of 0 and 100 μM. After 24 h, the cytoplasmic and nuclear protein were obtained using the NE‐PEP reagents kit (Thermo Fisher Scientific). After extraction, the expression of β‐catenin was measured in cytoplasmic and nuclear extracts using Western blot.

### 2.7 Statistical Analysis

The statistical significance of the results of this experiment was analyzed using GraphPad Prism Version 5.0 software (GraphPad, San Diego, CA, USA). The experimental results were expressed as mean ± standard error of the mean (SEM), and statistical significance for cell proliferation and apoptosis was tested by the Bonferroni test through two‐way analysis of variation (ANOVA). Statistical significance was defined as *p* < 0.05. Each experiment was performed at least three times independently.

## 3 Results

### 3.1 Effects of SFN on the Growth of Mia PaCa‐2 Cells

**FIGURE 1:**
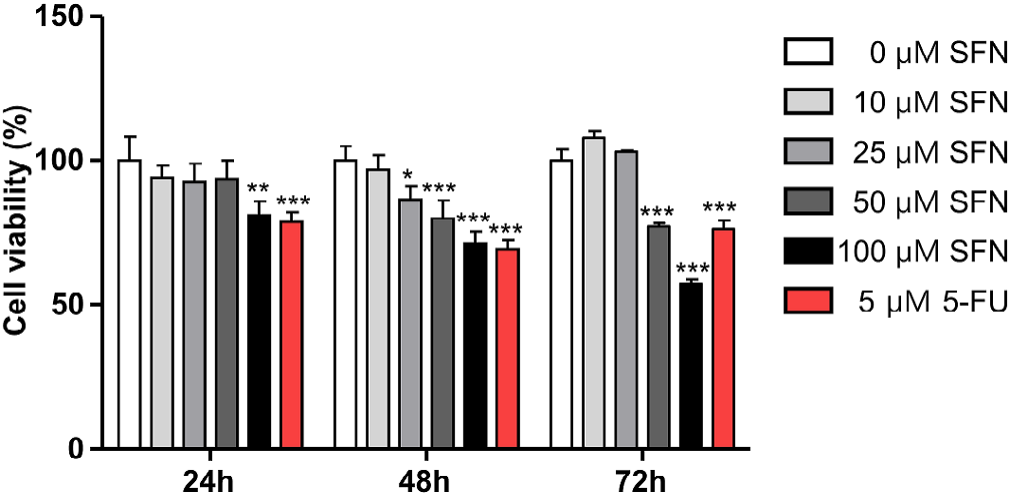
Effect of sulforaphane on the growth of Mia PaCa‐2 cells. Mia PaCa‐2 cells were seeded at 1 × 10^4^ cells/200 μL/well and were treated for 24, 48, and 72 h with sulforaphane (SFN) at concentrations of 0, 10, 25, 50, and 100 μM. 5‐Fluorouracil (5‐FU) was used as a positive control. The Celltiter 96 aqueous one solution cell proliferation assay kit was used to determine cell viability. Each experiment was performed at least three times independently. Statistical significance was based on the difference compared with 0 μM SFN by two‐way analysis of variance (ANOVA), followed by the Bonferroni test (*n* = 3) (**p < 0.01, ***p < 0.001).

First, the effects of SFN on the proliferation of Mia PaCa‐2 cells were measured. At the 24 h treatment with 100 μM SFN (81.11% ± 4.85%), the cell viability decreased by approximately 19% compared with 0 μM SFN (100.00% ± 8.33%). When 100 μM SFN was treated for 48 or 72 h, the proliferation was inhibited by approximately 29% (71.06% ± 4.35%) or 43% (57.22% ± 3.08%), respectively (Figure 1) (*p* < 0.001). These results indicate that SFN inhibits the cell proliferation of Mia PaCa 2 cells. In the subsequent experiments, the maximum concentration of SFN was set at 100 μM for 24 h.

### 3.2 Effects of SFN on the Apoptosis of Mia PaCa‐2 Cells

**FIGURE 2:**
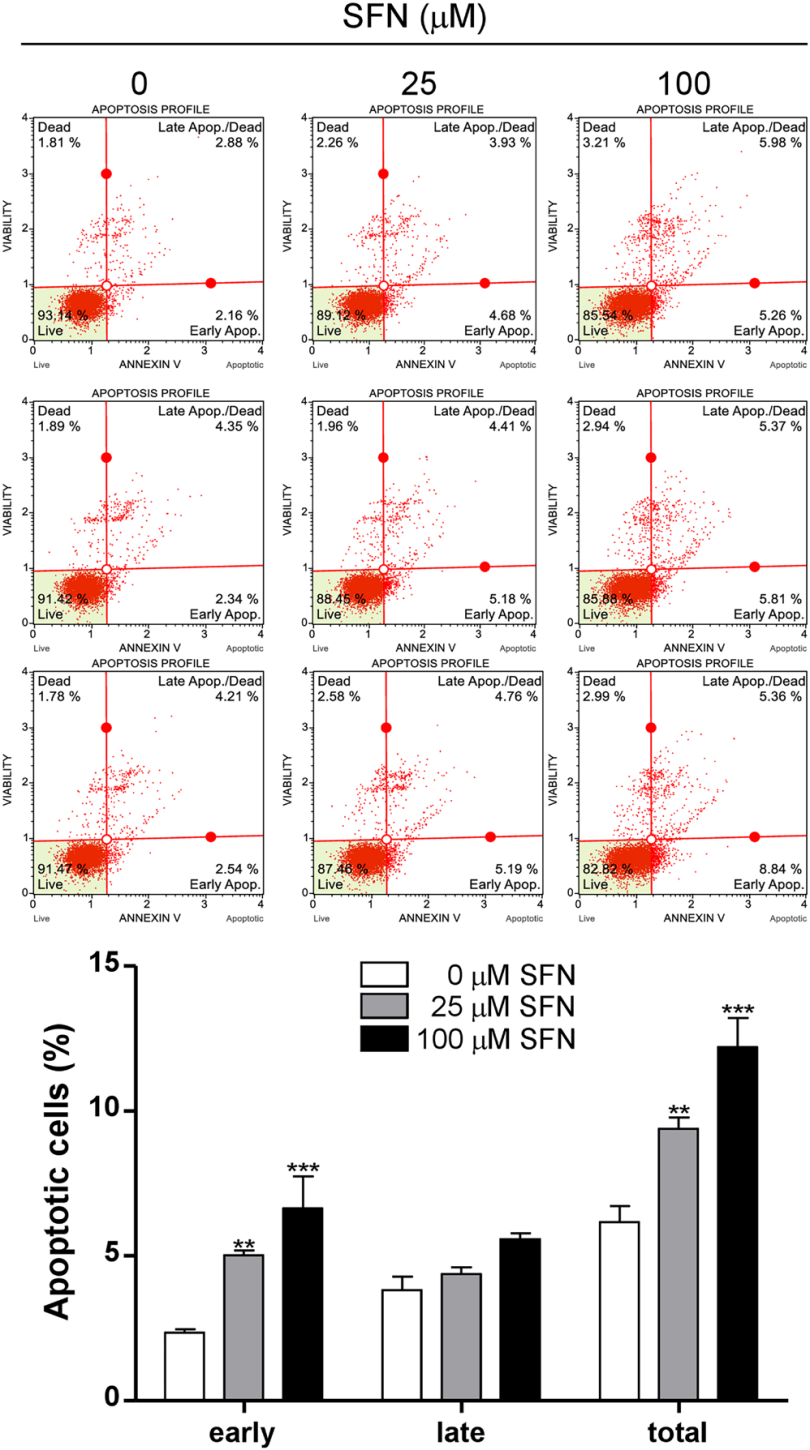
Effect of sulforaphane on the apoptosis of Mia PaCa‐2 cells. Mia PaCa‐2 cells were seeded at 5 × 10^4^ cells/mL/well and were treated for 24 h with sulforaphane (SFN) at concentrations of 0, 25, and 100 μM. The Muse Annexin V & Dead Cell Reagent was used to determine apoptosis. Each experiment was performed at least three times independently. Statistical significance was based on the difference compared with 0 μM SFN by two‐way ANOVA, followed by the Bonferroni test (*n* = 3) (**p < 0.01, ***p < 0.001).

Because SFN inhibited cell proliferation, the effects of SFN on apoptosis were evaluated through Annexin V and propidium iodide (PI) staining [14]. When 100 μM SFN was treated to Mia PaCa 2 cells for 24 h, the early apoptotic cells (Annexin V^+^‐PI^−^) were 6.64% ± 1.11%, whereas at 0 mM SFN, it was 2.35% ± 0.11% (***p < 0.001). The late apoptotic cells (Annexin V^+^‐PI^+^; to 5.57% ± 0.21%) increased at 100 mM SFN compared to 3.81% ± 0.47% at 0 μM SFN (***p < 0.001). The total apoptotic cells were increased from 6.16% ± 0.56% to 12.21% ± 1.00% by treatment with 100 μM SFN (Figure 2) (***p < 0.001). These data suggest that SFN induces apoptosis.

### 3.3 Effects of SFN on GSK‐3β and β‐Catenin Expression

**FIGURE 3:**
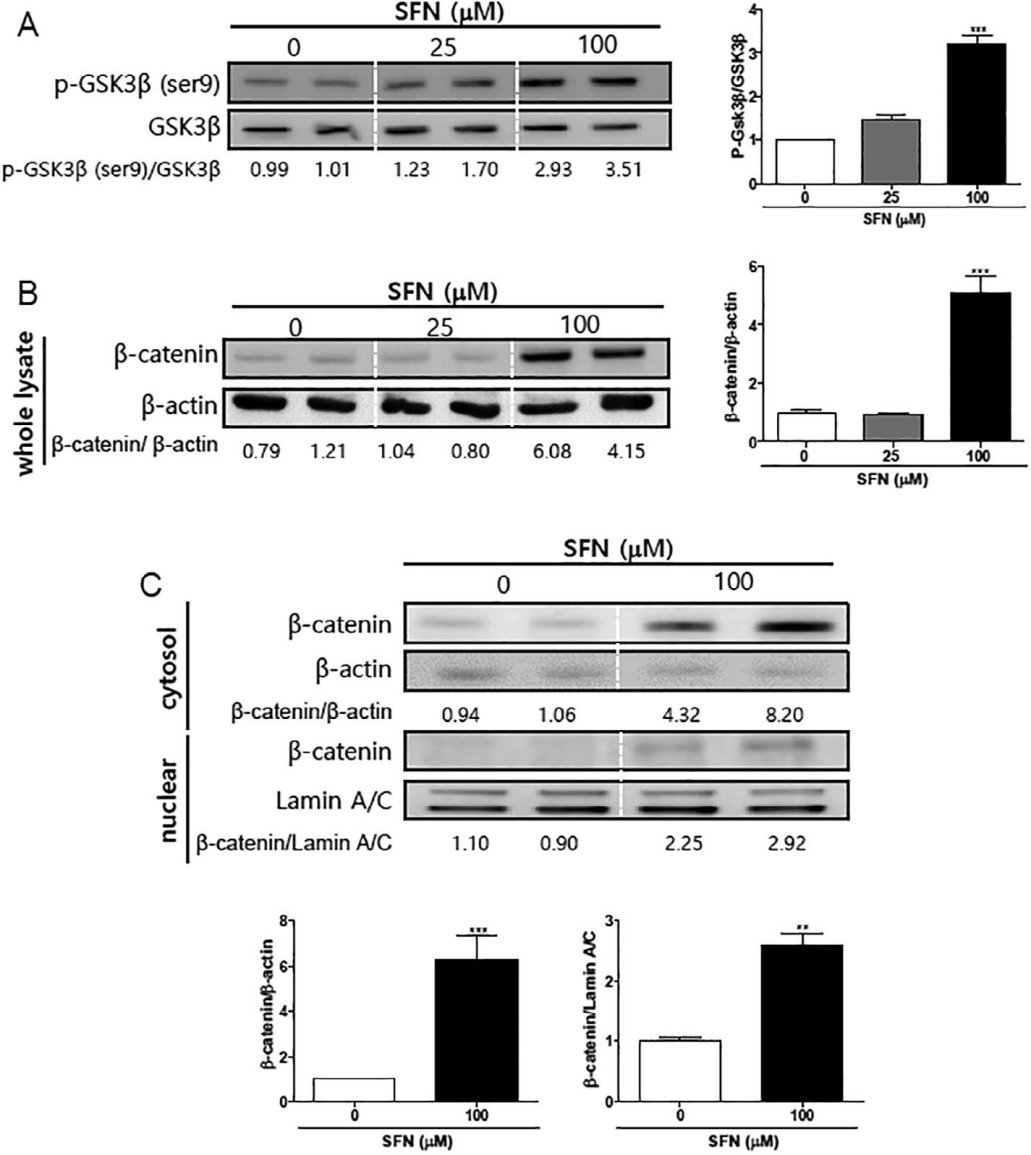
Effects of sulforaphane on GSK‐3β and β‐catenin expression. (A, B) Mia PaCa‐2 cells were seeded at 4 × 10^4^ cells/3 mL/well and were treated for 24 h with sulforaphane (SFN) at concentrations of 0, 25, and 100 μM. (C) Mia PaCa‐2 cells were seeded at 1 × 10^6^ cells/5 mL/well and were treated for 24 h with SFN at concentrations of 0 and 100 μM. Protein expressions of p‐GSK‐3β (Ser9), GSK‐3β, β‐catenin, and β‐Actin were measured using Western blot analysis. Relative intensities were divided by the dentition of the GSK‐3β or β‐Actin or Lamin A/C bands, respectively. Each experiment was performed at least three times independently. (A and B) Statistical significance was based on the difference compared with 0 μM SFN by one‐way ANOVA, followed by the Dunnett's test or (C) Student's t‐test (*n* = 3) (***p < 0.001).

Because SFN inhibited growth and induced apoptosis in Mia PaCa‐2 cells, the effect of SFN on the phosphorylation of GSK‐3β, which plays a significant role in pancreatic cancer progression [16, 17], was tested. The intensity of phosphorylation of GSK‐3β (Ser9)/GSK‐3β was significantly increased at 100 μM SFN (3.22 ± 0.17) compared with at 0 μM SFN (1.00 ± 0.01) (Figure 3A; ***p < 0.001). This data suggests that SFN induces the inactivation of GSK‐3β by increasing the phosphorylation of GSK‐3β (Ser9).

In a mutant Kirsten rat sarcoma viral oncogene homolog (KRAS)‐like‐dependent human cancer cell line such as in Mia PaCa‐2 cells, the inactivation of GSK3‐β induces apoptosis via the accumulation of β‐catenin [18]. Because SFN inactivates GSK‐3β by inducing the phosphorylation of GSK‐3β (Ser9), we subsequently evaluated the effect of SFN on the expression of β‐catenin. Based on the results throughout the whole lysate, the intensity of β‐catenin/β‐actin was significantly increased from 1.00 ± 0.12 (0 μM SFN) to 5.12 ± 0.56 (100 μM SFN) (Figure 3B; ***p < 0.001). We also analyzed the effects of SFN on the expression of β‐catenin in the cytoplasm and nuclear fractions. The expression of β‐catenin was increased from 1.00 ± 0.03 (0 μM SFN) to 6.26 ± 1.12 (100 μM SFN) (***p < 0.001) in the cytoplasmic fraction, and from 1.00 ± 0.06 (0 μM SFN) to 2.59 ± 0.19 (100 μM SFN) in the nuclear fraction (Figure 3C; **p < 0.01). These results suggest that the inactivation of GSK‐3β by treatment with SFN accumulates the β‐catenin in KRAS mutant cells such as Mia PaCa 2 cells.

### 3.4 Effects of SFN on NF‐κB Expression in Mia PaCa‐2 Cells

**FIGURE 4:**
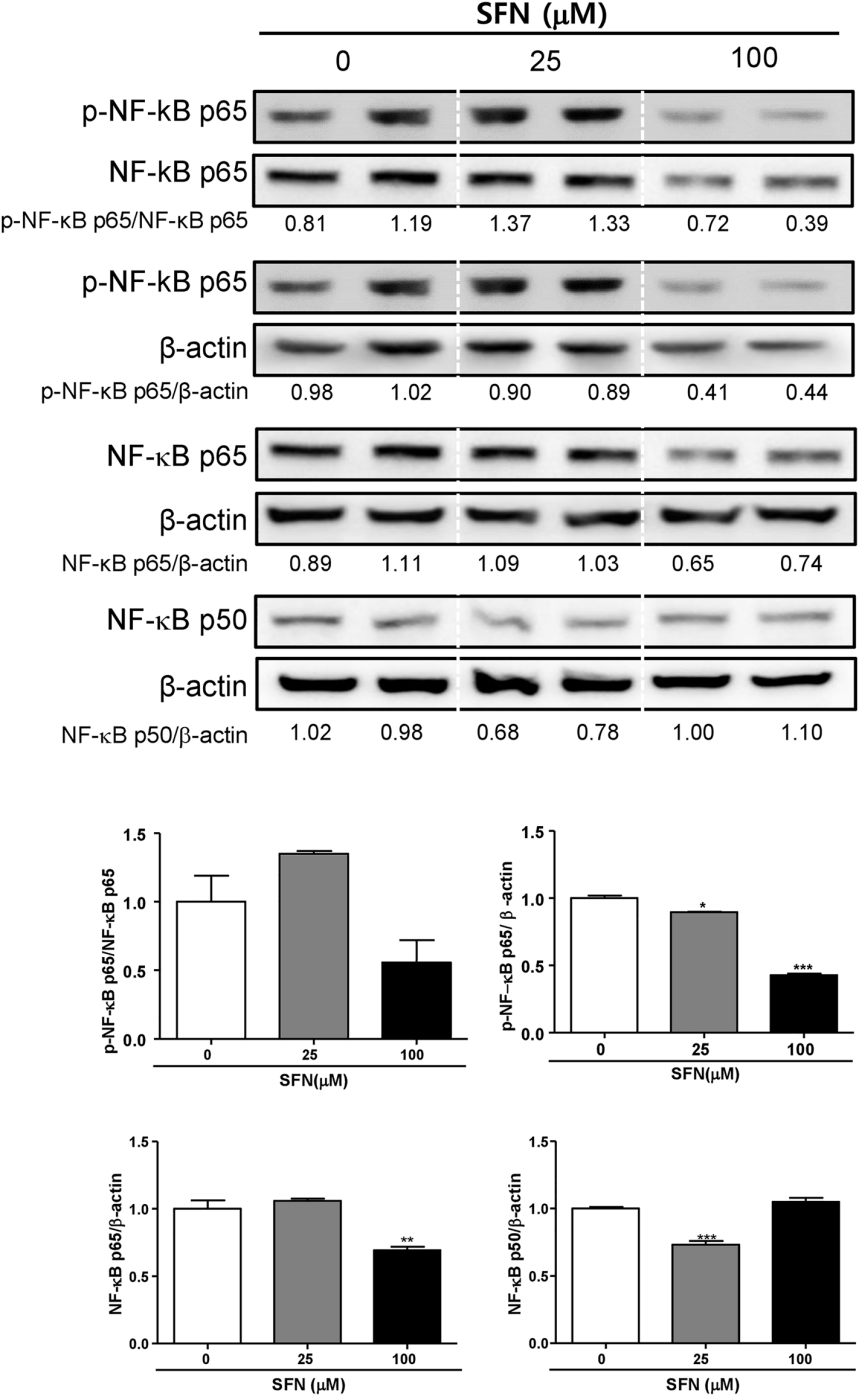
Effects of sulforaphane on NF‐κB expression in Mia PaCa‐2 cells. Mia PaCa‐2 cells were seeded at 4 × 10^4^ cells/3 mL/well and were treated for 24 h with sulforaphane (SFN) at concentrations 0, 25, and 100 μM. Protein expression of NF‐κB p65, NF‐κB p50, and β‐Actin were measured using Western blot analysis. Relative intensities were divided by the dentition of the β‐Actin bands. Each experiment was performed at least three times independently. Statistical significance was based on the difference compared with 0 μM SFN by one‐way ANOVA, followed by Dunnett's test (*n* = 3) (**p < 0.01, ***p < 0.001).

The activation of GSK‐3β in PDAC positively regulates NF‐κB transcription activity [17]. GSK‐3β promotes constitutive NF‐kB signaling, which is important in cancer cell survival and growth [19]. Because SFN induces the inactivation of GSK‐3β, we evaluated the expression of NF‐κB. Treatment of 100 μM SFN for 24 h significantly decreased the expression of the p65 subunit of NF‐κB from 1.00 ± 0.06 to 0.70 ± 0.03 (**p < 0.01), while the expression of the other NF‐κB subunit p50 was unaffected (0 μM SFN 1.00 ± 0.01; 100 μM SFN 1.05 ± 0.03). In addition, the expression of the p65 subunit of p‐NF‐κB was also reduced at 100 μM SFN(0.43 ± 0.02) compared with at 0 μM SFN (1.00 ± 0.02; Figure 4). These data suggest that SFN inhibits the expression of NF‐κB.

### 3.5 Effects of SFN on cMyc Expression in Mia PaCa‐2 Cells

**FIGURE 5:**
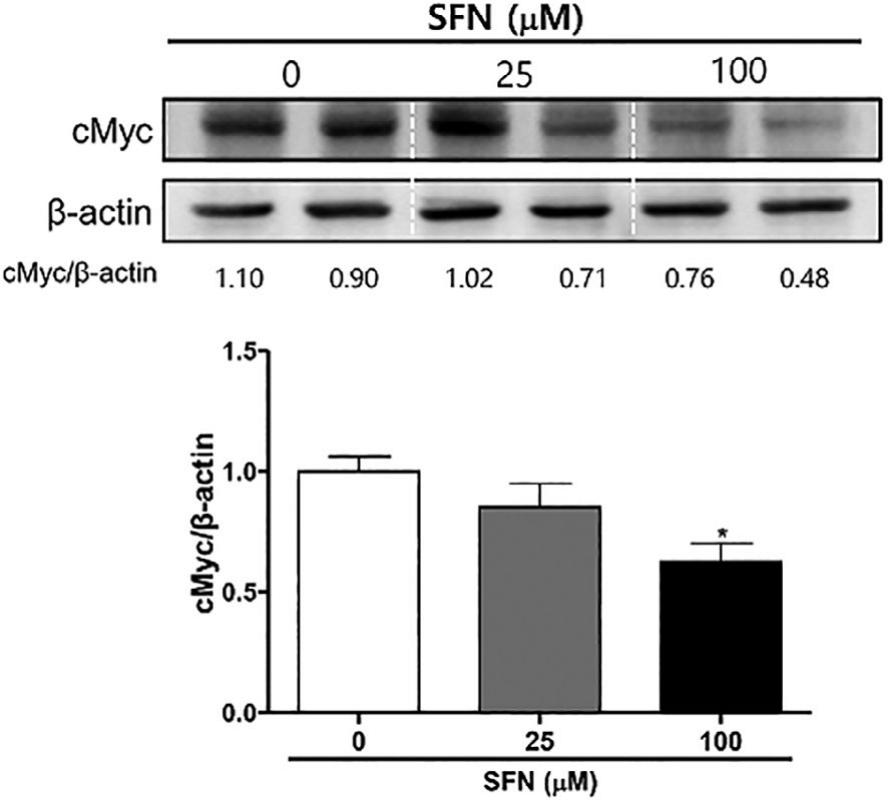
Effects of sulforaphane on cMyc expression in Mia PaCa‐2 cells. Mia PaCa‐2 cells were seeded at 4 × 10^4^ cells/3 mL/well and were treated for 24 h with sulforaphane (SFN) at concentrations of 0, 25, and 100 μM. Protein expression of cMyc and β‐Actin were measured using Western blot analysis. Relative intensities were divided by the dentition of the β‐Actin bands. Each experiment was performed at least three times independently. Statistical significance was based on the difference compared with 0 μM SFN by one‐way ANOVA, followed by Dunnett's test (*n* = 3) (*p < 0.05).

GSK‐3β inhibition decreases the expression of NF‐κB and NF‐κB‐mediated expression of cMyc in pancreatic cancer, thereby reducing tumor growth [20]. Because SFN treatment decreased NF‐κB p65, we evaluated the effects of SFN on the expression of cMyc. Treatment of 100 mM SFN (0.62 ± 0.08) suppressed the expression of cMyc compared with 0 μM SFN (1.00 ± 0.06) (*p < 0.05, Figure 5). These data suggest the possibility that SFN inhibits cMcy by suppression of the GSK‐3β/NF‐κB pathway.

### 3.6 Effects of SFN on the Expression of Apoptosis‐Related Proteins

**FIGURE 6:**
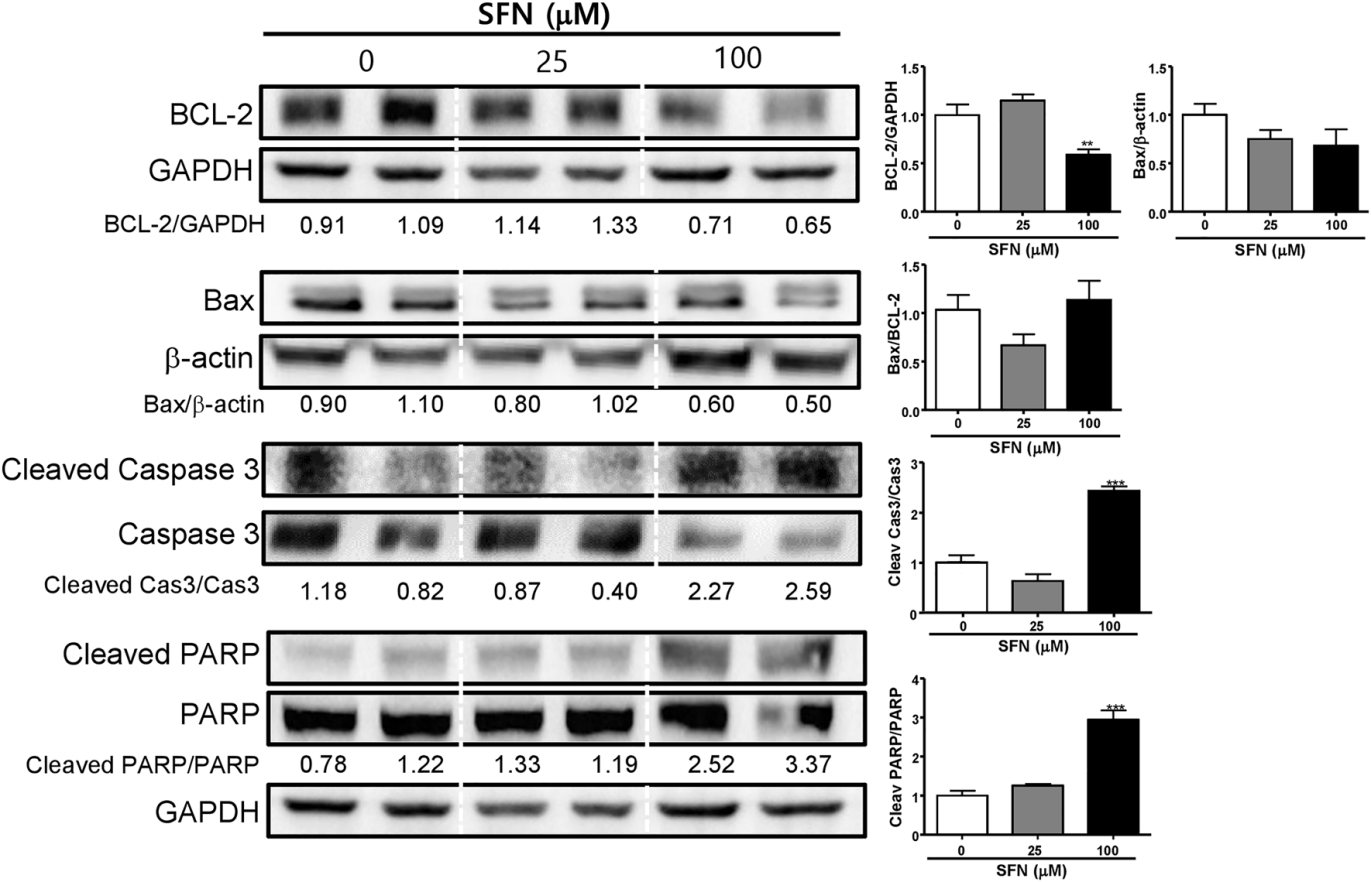
Effects of sulforaphane on the expression of apoptosis‐related proteins. Mia PaCa‐2 cells were seeded at 4 × 10^4^ cells/3 mL/well and were treated for 24 h with sulforaphane (SFN) at concentrations of 0, 25, and 100 μM. Protein expression of BCL‐2, BAX, cleaved caspase 3, caspase 3, cleaved PARP, PARP, and GAPDH were measured using Western blot analysis. Relative intensities were divided by the dentition of the GAPDH bands. Each experiment was performed at least three times independently. Statistical significance was based on the difference compared with 0 μM SFN by one‐way ANOVA, followed by the Dunnett's test (*n* = 3) (*p < 0.05, **p < 0.01, ***p < 0.001).

GSK‐3β facilitates the signal inducing the upregulation of the pro‐survival molecule BCL‐2 [16]. Additionally, NF‐κB is a transcription factor inducing BCL‐2 expression [21]. Hence, we evaluated the effect of SFN on the expression of BCL‐2. By treatment with 100 μM SFN for 24 h, the expression of BCL‐2 was decreased (0 μM SFN; 1.00 ± 0.11 vs. 100 μM SFN; 0.59 ± 0.06) (**p < 0.01, Figure 6). However, the expression of BAX, the pro‐apoptotic protein [22], was not significantly changed by treatment with 100 μM SFN (0.68 ± 0.17). Also, the intensity of BAX/BCL2, a known determinant of apoptotic sensitivity in response to apoptotic stimuli [23], was not significantly increased from 1.00 ± 0.15 (0 μM SFN) to 1.14 ± 0.20 (100 μM SFN).

Because SFN reduced the expression of BCL‐2, an anti‐apoptotic factor, we subsequently measured the effects of SFN in the activation of caspase‐3, which is responsible for nuclear alterations in apoptosis and is considered the main effector of caspase [24].

The intensity of cleaved caspase‐3/caspase‐3 was significantly increased from 1.00 ± 0.10 at 0 μM SFN to 2.43 ± 0.09 at 100 μM SFN (***p < 0.001, Figure 6). Poly (ADP‐ribose) polymerase (PARR) is a nuclear enzyme that has a well‐characterized role in base excision repair [25]. The level of cleaved PARP/PARP was increased from 1.00 ± 0.13 at 0 μM SFN to 2.95 ± 0.25 at 100 μM SFN (***p < 0.001, Figure 6). These results indicate that SFN induces apoptosis by increasing apoptosis‐related proteins.

## 4 Discussion

Mia PaCa‐2 cells are a human pancreatic adenocarcinoma cell line characterized by a poor degree of differentiation potential, high to moderate degree of resistance, high colony‐forming capacity, and high spheroid‐forming capacity compared to other cell lines [11], which are also the main features of tumor‐initiating cells involved in recurrence and drug resistance in pancreatic cancer. Li et al. reported that the value of 50%‐maximal inhibitory concentration (IC_50_) value was 10–15 μM when SFN was treated for 72 h in human pancreatic cancer cell lines [12]. Roy et al. reported that 1 μM SFN had an inhibitory effect on cell proliferation for 48 h treated with SFN in Mia PaCa‐2 cells [10]. Thakkar et al. reported that the value of IC_50_ was 10.7 μM in Mia PaCa‐2 cells after SFN treatment for 72 h [26]. However, in our study, the value of IC_50_ for SFN was 111.0 μM when SFN was treated for 72 h. Although the effect of SFN in this study is minimal, SFN has an inhibitory effect on the proliferation of Mia PaCa‐2 cells, which is consistent with the findings of previous studies [10, 12, 26]. Therefore, in the subsequent experiments, the maximum concentration of SFN was set at 100 μM for 24 h.

The staining method using Annexin V and PI can detect and quantify apoptotic and necrotic cells [15]. Annexin V, 36‐kDa calcium‐binding protein, can be used to detect phosphatidylserine (PS) that is exposed on the outside of apoptotic cells [15]. In this study, SFN induced early apoptosis at 25 and 100 μM concentrations (Annexin V^+^‐PI^−^). Early in the apoptotic pathway, molecules of PS on the cell surface are translocated to the outer surface of the cell membrane where Annexin V can readily bind them. In early apoptotic cells, the plasma membrane is normal, but because there is only movement of PS, Annexin V is positive [15]. Therefore, when SFN was treated with 25 μM for 24 h, it induced early cell death, but there was not enough time and concentration to decrease the number of viable cells. In the previous study, Roy's group reported that SFN induced apoptosis in PANC‐1 and AsPC‐1 cells [11]. Kallifatidis et al. found that SFN induced apoptosis through the protein kinase C (PKC) pathway [12]. In a study by Thakkar et al. SFN showed 13% cell death in Mia PaCa‐2 cells after 48 h treatment with 5 μM SFN [14]. These results suggest that SFN induces apoptosis in Mia PaCa‐2 cells.

GSK‐3β is a serine/threonine kinase that plays a role in the development of pancreatic ductal adenocarcinoma (PDAC) by cell proliferation, survival, invasion, metastasis, and resistance to chemotherapy [16, 17]. GSK‐3β is commonly overexpressed in various types of cancer such as pancreatic cancer [15]. Pancreatic cancer cell lines have decreased phosphorylation (Ser9), suggesting that GSK‐3β is largely in an activated state in PDAC to drive various protumorigenic processes [17]. PDAC with low Ser9 phosphorylation of GSK‐3β inflicts a negative prognosis owing to sustained tumor‐promoting signals [27]. Therefore, the induction of Ser9 phosphorylation of GSK‐3β can be a therapeutic target for identifying the chemotherapy agent.

β‐catenin was the substrate of GSK‐3β. The Wingless Int‐1 homolog (Wnt)/β‐catenin pathway is a key mechanism in many cellular processes. It begins with the binding of a Wnt ligand to its receptor, which inhibits the activity of GSK‐3β, leading to the accumulation of β‐catenin in the cytosol [27]. Once in the cytosol, β‐catenin is transported to the nucleus, where it interacts with the T‐cell factor/lymphoid enhancing factor (TCF), a transcription factor, and promotes the transcription of genes that regulate cell proliferation, differentiation, apoptosis, migration, infiltration, and tissue homeostasis [27, 28]. In general, GSK‐3β suppresses β‐catenin activation through proteasome targeting while the inhibition of GSK‐3β allows accumulation of β‐catenin followed by nuclear translocation and β‐catenin‐dependent gene transcription [13]. In the KRas mutant cell, Mia Paca 2 cells, the inactivation of GSK3‐β is known to induce apoptosis via the accumulation of β‐catenin [18]. Because in Figure 3a, the treatment of SFN inactivates GSK‐3β by phosphorylation of Ser9, we evaluated the expression of β‐catenin in the whole lysate and fractions. Indeed, SFN treatment induced the accumulation of β‐catenin. KRas, the most frequently mutated Ras gene, confers resistance to therapy in pancreatic cancer. Patients with mutant KRas cancers have poor prognosis, increased tumor aggressiveness and metastasis, and are less likely to respond to chemotherapy and targeted therapies [29]. In Mia PaCa‐2 cells, the suppression of GSK‐3β inhibits mutant KRas‐dependent tumor growth and the depletion of GSK‐3β increases β‐catenin levels and forces the expression of β‐catenin mutants that lack GSK3 phosphorylation sites and are thus insufficient to induce apoptosis. These results suggest that in mutant KRas‐dependent human tumors such as pancreatic cancer, GSK3 has pro‐survival activity, whereas β‐catenin has pro‐apoptotic activity [29].

GSK‐3β is a key regulator of NF‐κB in pancreatic cancer cells, which controls the expressions of genes governing cell proliferation, apoptosis, inflammation, angiogenesis, infiltration, and drug resistance [30]. The inhibition of GSK‐3β results in the suppression of pancreatic tumor growth and survival of pancreatic cancer cells related to NF‐κB [30]. Using an electrophoretic mobility shift assay (EMSA), SFN inhibited the binding of NF‐κB dimers in Mia PaCa‐2 cells [11]. The same study also reported that SFN increased apoptosis in cRel knock‐down Mia PaCa‐2 cells [11]. A previous study has shown that SFN could induce Ser9 phosphorylation in GSK‐3β and inhibit the expression of NF‐κB p65 [16]. These results suggest that SFN inhibits the expression of NF‐κB through inactivation of GSK‐3β.

cMyc oncoprotein has been implicated in the pathogenesis of pancreatic cancer such as cellular growth, division, differentiation, survival, apoptosis, and angiogenesis [13, 20, 31]. GSK‐3β inhibition decreases the expression of NF‐κB and NF‐κB‐mediated expression of cMyc in pancreatic cancer, thereby reducing tumor growth [20]. GSK‐3β is a critical regulator of NF‐κB nuclear activity, suggesting that inhibition of GSK‐3β could be effective in the treatment of a wide variety of tumors with constitutively active NF‐κB [32]. Mutant KRas activates Raf/MEK/ERK pathway to upregulate cMyc. NF‐κB can activate the transcription of the cMyc promoter, suggesting that NF‐κB promotes cell cycle entry through the upregulation of cMyc expression [14]. In our data, SFN inhibited the expression of NF‐κB p65 and cMyc. These data suggest that SFN inhibits cMyc through inhibition of GSK‐3β and NF‐κB p65.

Inhibition of GSK‐3β causes sensitization to apoptosis induced by tumor necrosis factor‐related apoptosis‐inducing ligand in PDAC [17]. Anti‐apoptosis proteins such as BCL‐2 are up‐regulated via the activation of the NF‐κB pathway during the emergence of chemoresistance in invasive pancreatic cancer [33]. Consistent with our data, 9‐ING‐41, a small molecule inhibitor of GSK‐3β, has demonstrated anti‐tumor activity in patient‐derived xenograft mice (PDX) of various human cancers including PDAC by inhibiting the NF‐κB target genes such as BCL‐2 [16].

Caspase 3 is a cysteine protease and has a key role in apoptosis [24]. Cleavage of PARP by caspase‐3 inactivates the DNA repair ability of PARP [25]. When PARP is cut by caspase‐3, its ability to repair DNA is inhibited [25]. In Mia PaCa‐2 cells with abnormal KRas, the inhibition of GSK‐3β was found to induce the activation of caspase‐3 and PARP fragmentation [17]. In Mia PaCa‐2 cells, SFN was shown to induce caspase‐3 activity [10, 11]. These results indicate that SFN induces apoptosis through the regulation of BCL2, caspase‐3, and PARP.

**FIGURE 7:**
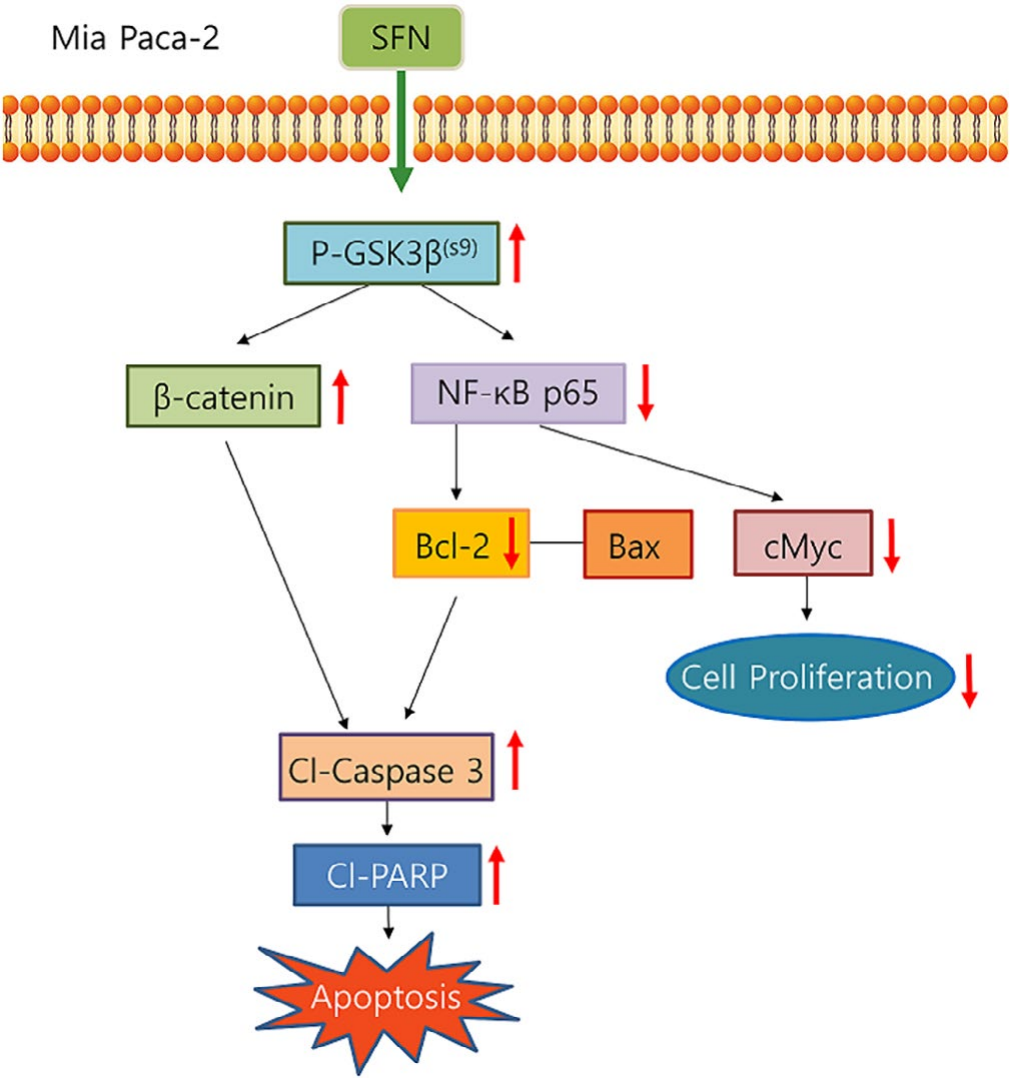
Anti‐cancer effect of sulforaphane. Sulforaphane (SFN) inhibited the activity of GSK‐3β by phosphorylation of Ser9 of GSK‐3β. Inactivation of GSK‐3β increases apoptosis by β‐catenin/caspase‐3/PRAR pathway and NF‐κB/BCL‐2 pathway. In addition, SFN inhibits cell proliferation through inhibition of NF‐κB/cMyc.

In conclusion, SFN induces the inactivation of GSK‐3β by phosphorylation of Ser9. Inactivation of GSK‐3β increases apoptosis by the β‐catenin/caspase‐3/PRAR pathway and NF‐κB/BCL‐2 pathway. Also, SFN inhibits cell proliferation through inhibition of NF‐κB/cMyc (Figure 7). These data suggest that SFN has a potential anticancer effect.

## Author Contributions

**Min Ju Park:** writing – original draft, writing – review and editing, methodology, formal analysis, data curation, investigation. **Yoon Hee Kim:** writing – review and editing, writing – original draft, supervision, conceptualization, funding acquisition.

## Conflicts of Interest

The authors declare no conflicts of interest.

## Data Availability

This is an open‐access article under the terms of the Creative Commons Attribution License, which permits use, distribution and reproduction in any medium, provided the original work is properly cited.
